# The combination of melatonin implants and prostaglandin F2α improves lamb production in a late-autumn mating season

**DOI:** 10.1007/s11259-022-09990-9

**Published:** 2022-08-26

**Authors:** J. Barbanoj, J. A. Abecia

**Affiliations:** Departamento de Producción Animal Y Ciencia de los Alimentos, Instituto Universitario de Investigación en Ciencias Ambientales (IUCA), Universidad de Zaragoza, Miguel Servet, 177, 50013 Zaragoza, Spain

**Keywords:** Sheep, Melatonin, Prostaglandins

## Abstract

To determine the effect of the combination of melatonin implants and prostaglandin (PG) F2α on reproductive performance in the late breeding season (Dec at the northern hemisphere), 500 Lacaune ewes were divided into four groups. On day 0 (7 Nov), 150 ewes were treated with a melatonin (M) implant. From that group, 64 ewes (M + 1PGF group) were injected with 10-mg prostaglandin (PG) F2α 34 d after melatonin implantation (11 Dec). The remaining 86 ewes (M group) were treated with melatonin, only. Another group of 75 ewes (2PGF group) was treated with double injection of PGF2α (9 days between the first and second application) (2 and 11 Dec), and 75 non-treated ewes (C group) were the control group. The remaining 200 ewes of the flock were not considered in the study. Rams (n = 23) were introduced on 11 Dec. The percentage of prolificacy, lambing and fecundity rates were calculated. Lambing rate did not differ among groups (M: 79%; M + 1PGF: 78%; 2PGF: 69%; C: 71%). The M + 1PGF group had a higher % of prolificacy than the 2PGF group (*P* < 0.10) and the C group (*P* = 0.06) (M: 1.65 ± 0.07; M + 1PGF: 1.74 ± 0.09; 2PGF: 1.54 ± 0.08; C: 1.54 ± 0.07 lambs/lambing) (*P* < 0.05), and a higher fecundity than the 2PGF group (*P* < 0.05) and the C group (*P* < 0.10) (M: 1.30 ± 0.09; M + 1PGF: 1.36 ± 0.11; 2PGF: 1.07 ± 0.10; C: 1.08 ± 0.09 lambs/ewe). Ewes implanted with melatonin had significantly higher prolificacy (1.69 ± 0.06 lambs/lambing) (*P* < 0.05) and fecundity (1.33 ± 0.07 lambs/ewe) (*P* = 0.01) than did ewes that did not receive melatonin (1.54 ± 0.04 and 1.08 ± 0.04, resp.). In conclusion, melatonin implants increased the number of lambs born per ewe in a late-autumn mating season, and the effect was greatest if it was given in combination with PGF2α administration at ram introduction.

Reproduction in sheep can be artificially controlled by administering exogenous hormones, which imitates the physiological chain of events of the natural sexual cycle (reviewed by Abecia et al. 2011, 2012). Melatonin implants have been used to advance the breeding season of anestrous ewes. The implants cause a short-day-like response by lengthening the duration of the melatonin signal (Malpaux et al. 1997). Although melatonin implants generally are used in the non-breeding season in Mediterranean sheep (Kouimtzis et al. 1989; Mura et al. 2017), they can be used in the late breeding season to increase the number of ewes lambing (Palacios et al. 2010). On the other hand, the beneficial effects of melatonin on embryo quality have been reviewed (Abecia et al. 2018), both during the breeding and the anoestrous seasons.

The prostaglandin (PG) F2α is a luteolytic factor in ruminants (Fierro et al. 2013), and the administration of exogenous PGF2α or its analogues can induce controlled luteolysis in the ovulatory season as a means of synchronizing estrus.

Given the useful effects of exogenous PGF2α in the breeding season, the aim of this study was to determine whether a combination of melatonin implants and PGF2α might provide a useful hormonal treatment for increasing lamb production in the December mating season, late autumn in the northern hemisphere.

## Material and methods

In early Nov 2020, 500 meat-line adult Lacaune dry ewes (2–7 years-old), which were weaned from their previous lambing at least three months ago, were randomly divided into four group: on 7 Nov (day 0), 150 ewes were given a subcutaneous melatonin (M) implant (18 mg melatonin, Melovine, CEVA Salud Animal, Barcelona, Spain) at the base of the left ear. From that group, 64 ewes (M + 1PGF group) received a 10-mg injection of PGF2α (2 ml Prosyl, 0.2 mg/kg LW for a 50 kg-standard ewe; CEVA Salud Animal, Barcelona, Spain) 34 d after melatonin implantation (11 Dec). The remaining 86 ewes (M group) were treated with melatonin, only. Another group of 75 ewes (2PGF group) received PGF2α injections on 2 and 11 Dec, and 75 non-treated ewes (C group) were used as the control. Animals were kept in a single flock, and adult Lacaune rams (n = 23) were introduced on 11 Dec for two months, with diurnal grazing, and housed in a stable at night. They were supplemented with a commercial pellet diet (0.5 kg/ewe/day) from the last month of pregnancy.

In the lambing season (4 May—22 Jun), prolificacy (number of lambs born / number of ewes lambing), lambing rate (number of ewes that lambed / number of ewes exposed to the ram) × 100), and fecundity (number of lambs born / number of ewes exposed to the ram) were calculated for each group. Differences in lambing rate were evaluated statistically by the X^2^ test, and the effects of the treatments on prolificacy and fecundity were evaluated statistically by a multifactorial ANOVA in the General Linear Model (GLM) procedure of SPSS (v.26), in a model that included melatonin and PGF2α treatment of the ewes, and their interaction. The cumulative proportion (%) of ewes lambing per day (lambing curve), and the number of ewes lambing within 10-d intervals were calculated.

## Results and discussion

Lambing rate did not differ significantly among groups (Table 1). The use of melatonin significantly affected both prolificacy and fecundity (P < 0.05), but the inclusion of PGF in the treatment did not (Table 1). The M + 1PGF group had a higher mean prolificacy than the 2PGF (*P* < 0.10) and the C (*P* < 0.05) groups, and had a higher fecundity than the 2PGF (*P* < 0.05) and the C (*P* < 0.10) groups (Table 1). Ewes implanted with melatonin (M and M + 1PGF groups), had significantly higher prolificacy (1.69 ± 0.06 lambs/lambing) (*P* < 0.05) and fecundity (1.33 ± 0.07 lambs/ewe) (*P* = 0.01) than ewes that did not receive melatonin (2PGF and C groups) (1.54 ± 0.04 and 1.08 ± 0.04, for prolificacy and fecundity, resp.); however, ewes that were treated with PGF2α (M + 1PGF and 2PGF groups) and those that were not treated with PGF2α (M and C groups) did not differ significantly in prolificacy (1.63 ± 0.06 vs. 1.60 ± 0.04) or fecundity (1.23 ± 0.06 vs. 1.15 ± 0.07).

**Table 1:** Proportion (%) of lambing (number of ewes lambed/number of ewes of the group), mean (± S.E.M.) prolificacy (number of lambs born/lambing), and fecundity (number of lambs born/ewe) of meat-line Lacaune ewes hat received either one melatonin implant 34 d before ram introduction (M), one melatonin implant plus one dose of prostaglandin (PG) F2α 34 d after melatonin implantation (M + 1PGF), two doses of PGF 9 and 0 days before ram introduction (2PGF), or control, non-treated ewes (C)

	M	M + 1PGF	2PGF	C	Effect M	Effect PGF
N	86	64	75	75		
Lambing rate (%)	79	78	69	71		
Prolificacy (lambs/lambing)	1.65 ± 0.07^ab^	1.74 ± 0.09^b^	1.54 ± 0.08^a^	1.54 ± 0.07^a^	*P* = 0.047	*P* = 0.636
Fecundity (lambs/ewe)	1.30 ± 0.09^ab^	1.36 ± 0.11^b^	1.07 ± 0.10^a^	1.08 ± 0.09^a^	*P* = 0.016	*P* = 0.841

^a,b^ within a row, means without a common superscript differ (*P* < 0.05)

**Fig. 1:**
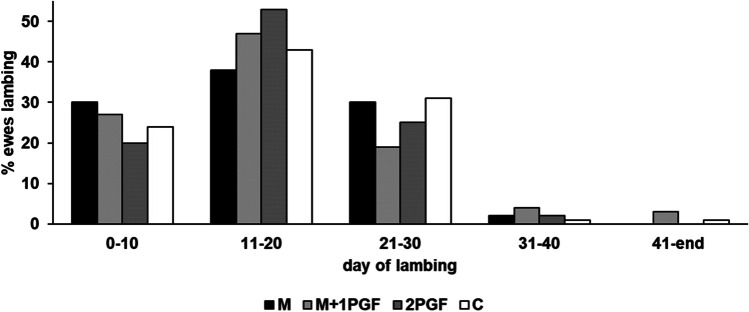
Proportion (%) of ewes that lambed within 10-d intervals of meat-line Lacaune ewes that received either melatonin implants 34 d before ram introduction (M), melatonin implants plus one dose of prostaglandin (PG) F2α 34 d after melatonin implantation (M + 1PGF), two doses of PGF 9 and 0 days before ram introduction (2PGF), or control, non-treated ewes (C)

All four treatment groups of ewes reached 80% of lambing within three weeks, although about half of the animals in the two groups that received PGF2α, parturitions were concentrated between days 10 and 20 of the lambing period (Fig. 1).

The positive effect of exogenous melatonin on lamb production in a breeding-season mating period was the most remarkable result of this experiment. Forcada et al. (2002) demonstrated the efficacy of melatonin implants inserted immediately after the winter solstice, which either advanced the LH secretion of estradiol-treated, ovariectomized ewes in the absence of males, or improved reproductive parameters under field conditions in the subsequent mating period. The increase in the number of lambs born per lambing among melatonin-treated sheep during the breeding season might have been caused by an increase in embryo survival, either through improvement in luteal function, a reduction in antiluteolytic mechanisms, enhancement in embryo quality, or effects on mechanisms involved in fertilization from ovulation to maternal recognition (reviewed by Abecia et al. 2018). Ewes in the melatonin-treated group that received a single dose of PGF2α at ram introduction produced 26% more lambs than did those in the control group and 27% more than did those in the groups that received two doses of PGF2α. The higher prolificacy of the M + 1PGF compared to that of the 2PGF group indicates that the melatonin treatment, rather than the PGF2α administration, was responsible for the increase in lambing.

Both of the protocols that involved PGF2α produced in some degree a lambing synchronization from day 10 to 20 of the lambing period, although it was expected that a concentration of lambings might occur at the beginning of the lambing period. The ram effect induces increases in LH secretion in the ovulatory season in cycling and in PGF2α-treated ewes (Contreras-Solís et al. 2009). In the conditions of our experiment, ewes either did not manifest estrus behaviour after the first LH surge of the oestrus cycle induced after the luteolysis produced by the prostaglandins, or the fertility of such estrus was low, which induced a new estrus 17 days later. Those phenomena might have been caused by a lack of synchrony in the LH surge with ram introduction or CL at the time of PGF injection.

In conclusion, melatonin implants increased the number of lambs born per ewe in a late-autumn mating period (December in the northern hemisphere), and that effect was greatest if it was given in combination with PGF2α administration at ram introduction.

## Data Availability

The datasets in this study are available from the corresponding author on reasonable request.
